# Optimized Power System Management Scheme for LSS PV Grid Integration in Malaysia Using Reactive Power Compensation Technique

**DOI:** 10.1002/gch2.201900093

**Published:** 2020-01-09

**Authors:** Mahesh Mohanan, Yun Ii Go

**Affiliations:** ^1^ School of Engineering and Physical Sciences Heriot‐Watt University Malaysia 62200 Putrajaya Malaysia

**Keywords:** FACTS, IEEE‐9 bus, LSS PV, PSS/E, PVSyst, reactive power compensation

## Abstract

A large‐scale solar photovoltaic system (LSS PV) aims to reduce the gap as Malaysia plans to shift electricity generation from conventional sources like fossil fuels to renewable energy sources. The government plans to increase renewable energy to 20% of the generation mix by 2025. The first and second round of Malaysia's LSS programme has 958 MW of PV projects to be realized by 2020. The third round of the LSS program goes for an aggregate capacity of 500 MW. Being an intermittent source of energy, the major complication is with grid integration of the LSS PV system into the national power grid. This research aims to identify an optimum power system management scheme for LSS in Malaysia to stabilize voltage fluctuations by utilizing IEEE bus configuration. The simulation and planning of network type is based on PSS/E and PVSyst. The expected outcome of this research is to develop a solution for LSS grid integration with minimal loss in the system and in accordance with electricity standards as per Malaysian grid code. Additionally, the harmony of incorporating power electronic devices for reactive power compensation is tested. This work can be stated as a reference model for utility provider in other countries having similar network and grid configuration.

## Graphical abstract

A large‐scale solar photovoltaic system (LSS PV) contributes significantly to Malaysia's energy transformation for electricity generation. One of the major complications is the integration of LSS PV with the national power grid. This research proposes an optimum power system management scheme that stabilizes voltage fluctuations using a reactive power compensation technique. This optimal solution exhibits minimal system loss, which complies with Malaysian grid code standard.

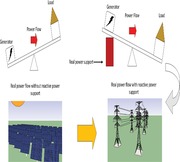

## 1 Introduction

The equatorial location of Malaysia has been an advantage for exploring the potential of large scale solar photovoltaic system (LSS PV) installations in the country. Solar power is the most preferred renewable energy source in Malaysia when compared to wind or other renewable energy sources. The statistics from the National Renewable Energy Policy and Action Plan (NREPAP) provides some insights about the potential trend of solar power installations in the country. The energy generation from renewables including solar PV, biogas, biomass, hydro and solid waste is expected to reach 11 227 GWh by 2020. Solar PV share from the total renewable energy generation would be around 1.7% or 194 GWh in 2020.^1^ The prediction for the solar energy generation by 2050 in Malaysia is at 46% of the total renewable energy generation. This accounts to 13 540 GWh of energy from solar PV.^2^ The LSS PV development in Malaysia under the Energy Commission has already completed 3rd cycle with an aggregate capacity of 500 MW.^3^ The names of the successful bidders responsible for the commissioning and operation of the LSS PV in Malaysia has been announced. Malaysia has pledged a commitment to reduce 45% of greenhouse gas (GHG) emissions by 2030 through the Intended National Determined Contribution (INDC) submitted to the United Nations Framework Convention on Climate Change (UNFCCC).^4^

### 1.1 Grid Connected PV Systems

The standards for grid integrated photovoltaic systems are described by the department of Standards Malaysia in MS: 1837. The specifications of the electronic components utilized for grid connected system are provided in the document. The schematic connection diagram accepted in Malaysia is determined in the Malaysian Standards MS: 1837.^5^ Another important component of the PV system is inverters. The technique of Maximum Power Point Tracking (MPPT) and the benefits of selecting inverter modules with MPPT feature were explained by P. Breeze.^6^

### 1.2 Technical Barriers to Large Scale Solar Grid Integration

The technical constraints faced in grid integration of LSS PV to the power grid are voltage variations, reactive power regulation, short circuit power deviations, voltage unbalance, power system instability, harmonic distortions and fluctuations at point of common coupling.^7^ The voltage quality issues are the main concern faced due to the intermittency of PV power output. The impacts of intermittent supply are voltage rise, voltage unbalance, voltage fluctuations and flickers on the grid connected network.^8^ The grid connected inverter combined with Proportional Integral (PI) controller was proposed to augment voltage curve in microgrids.^9^ The active and reactive power compensation was possible under unbalanced conditions at the point of common coupling. The method also witnessed reduced distortions under rapid load variations. The Continuation Power Flow (CPFLOW) based algorithm was suggested by W. Suampun to analyze the voltage stability of grid integrated systems and simulation was executed using IEEE‐14 bus system and IEEE‐39 bus system with varying loads. The research concluded that the PV penetration levels and the location of grid connected PV system play a crucial role in the stability of the grid.^10^

### 1.3 Power System Stability

Power system is a nonlinear network dependent on many parameters like generator outputs, transmission parameters, key operating specifications etc. which varies continuously.^11^, ^12^ The power system stability can be classified into steady state, transient and dynamic stability.^13^ An unstable system can lead to break down of the entire system or part of the system. The nature of fault or disturbance can be small or large scale ranging from short to long time when measured in timescale. The power quality enhancement with the implementation of multilevel inverter topology and then injecting power into the grid for hybrid solar and wind conversion system was investigated by Sharma et al. The method also had an advantage of maximum power extraction from the renewable energy sources.^14^ The enhancement of fault ride through capability during grid faults with revised protection control measures for DC link of grid connected PV system was proposed in agreement with grid code specifications. The over voltage at the DC link and the AC current at the common coupling point was prevented with the support of reactive power. This facilitates the power supply from the inverter during abnormal conditions of the power network.^15^

The load of the system including transmission losses are matched to the generation to keep the frequency constant, i.e., 50 Hz in Malaysia. The main analysis tool for the steady state operation is the power flow analysis through which voltages and power flow are regulated. This method is adopted for the planning studies and operation of high voltage transmission networks and low voltage distribution networks.^16^ A comparative study of voltage stability assessment with wind generator and solar PV generator was carried out by Youssef et al. on an IEEE‐14 bus system. The bus voltages during different contingency were analyzed and the most affected bus was determined.^17^

The reactive power compensation for reactive loads is provided to increase the overall efficiency of the grid network. The capacitor banks are installed usually in practice for VAR generation. The additional compensation devices can be avoided to a great extent by reactive power injection to the grid connected converters in the renewable energy generation system. The power transmission losses and the voltage dips relevant in the grids can be reduced by the reactive power compensation technique.^18^ FACTS devices are connected through series or shunt compensation in AC networks to increase power transfer capability and stability of the network. The device can also provide loss optimizations and manage congestions in the network.^19^ The reactance of the lines is controlled by series connected FACTS devices while the shunt connected devices are used for compensation in transmission lines. Series compensation is provided by Static Synchronous Series Compensator (SSSC) and Thyristor‐Controlled Series Capacitor (TCSC).^19^, ^20^, ^21^ The STATCOM and SVC are examples of shunt compensation devices. The FACTS controller offers to regulate the transmission of AC, increasing or decreasing power flow in specific branch and response to stability issues. The insufficiency in reactive power leads to voltage sags and required real power is not received at load through the transmission lines due to losses.

## 2 Malaysian Grid Code

The design and operation of the transmission system in Malaysia is specified through the transmission system performance characteristics in the grid code for peninsular Malaysia. The grid frequency range adopted in Malaysia is between 49.5 and 50.5 Hz for normal operation. The exceptional case can experience the operation of equipment within frequency limit of 47–52 Hz with predefined operation measures. The continuous operation happens from 47.5 to 52 Hz while the operation is restricted for 10 s every time frequency drops below 47.5 Hz.^22^

The next parameter of concern is the voltage variations defined for each type of transmission system and their tolerance from the nominal values. The nominal value of 500 KV network shall remain within ±5% for normal operating conditions. The abnormal operation can experience voltage deviations of 10% for a maximum of 15 min if deviation is positive.^22^ The 275 and 132 KV have a nominal value of ±10% for permissible variations in voltage. Any network below 132 KV allows a maximum deviation of ±6%.

The plant performance requirements are listed in the grid code for Peninsular Malaysia. The generating units must supply rated power output within a range of 0.85 power factor lagging and 0.95 power factor leading. The short circuit ratio at the very least of 0.5 is allowed for the generating units. The active power output must not be influenced by variations in voltage for normal operating range of generating units or power park module under steady state conditions. The reactive power output should be entirely available with the voltage range ±5% at 500, 275, and 132 KV and lower voltages.^22^

## 3 Methodology

### 3.1 Project Methodology

The research focuses on the assessment of a power system management framework for the LSS PV grid integration in Malaysia. The study was conducted by integrating the solar power generation bus to the IEEE‐9 bus test bed. The IEEE‐9 bus deployed is a standard test system in accordance with the Western System Coordinating Council (WSCC). The research has two primary evaluation performed throughout the project. The simulation for these two evaluations is performed using PVSyst version 6.8.1 and PSS/E by Siemens.

The first stage involves the study of the PV modules, configuration of modules, calculation of yearly energy yield, maximum energy produced, and maximum energy injected to the grid. PVSyst was used to model the solar system with input parameters specified in the data sheet for each PV module. The inverter output in AC rating was analyzed and fed as input to the PSS/E for power flow analysis.

The second stage utilizes the PSS/E by Siemens to integrate the solar generation plant with the IEEE‐9 bus test configuration to analyze the effects of voltage variation and reactive power demand. The best option for integrating the solar generation bus was examined.

The third and final stage was performance improvement using additional power electronics for the configuration options having large deviations on bus voltage levels. The enhancement in the operation of network with additional components was noted to compare the results.

### 3.2 Feasibility of Solar PV Power Plant

#### 3.2.1 Decision on Site

Malaysia receives abundant sunshine throughout the year and the irradiance measured in Malaysia suggests the feasibility of LSS PV. The annual irradiance received by the country is around 1900 KWh m^−2^.^23^ The characteristics of the location selected for the deployment of LSS PV power plant is one of the main factors that contribute to the energy output of the system. The Energy Commission under the Malaysian government has listed numerous sites suitable for LSS PV in Malaysia. In this study, one of the sites announced by the Energy Commission is considered to provide realistic results. The site chosen for this study is a 50 MW capacity grid connected LSS PV at Sepang, Selangor which falls under the jurisdiction of the Tenaga Nasional Berhad (TNB).^24^

#### 3.2.2 Solar Irradiation and Yearly Energy Yield

The power output of a PV system depends on the solar irradiation received on site rather than the temperature at site. The higher temperatures cause negative effects on the energy production as the performance of solar PV is inversely proportional to temperature. The research is limited to the annual energy yield and solar irradiation at Sepang, Selangor as the nearest station for meteonorm data was located at Sepang. Since the meteonorm data for Sepang was not available on the PVSyst database, the irradiation values were generated by the software. The monthly Global Horizontal Irradiation (GHI) was tabulated and the yearly GHI was 1610 KWh m^−2^.

#### 3.2.3 Power Network and Infrastructure

The maximum utilization of the generated energy is possible when the transmission losses are at minimal value. The solution can be achieved by transmitting energy from generation plant to distribution network located at shorter distance rather than longer distance. The long distance transmission can lead to increase in losses and in AC transmission, the impedance value of the transmission line plays a vital role. The location of LSS PV being Selangor has advantages due to the presence of a strong transmission and distribution network.

### 3.3 Solar PV Generation Profile

The assessment includes three monocrystalline PV modules and two thin film PV modules. These modules were chosen on the basis of local availability in Malaysia. Furthermore, the manufacturers like First Solar, Hanwha Qcells, and Longi Solar are approved local manufacturers by the SEDA Malaysia. The descriptions of the selected PV models are as listed in **Table** **1**.

**Table 1:** PV modules in Malaysia and their specifications

Manufacturer	Model No.	Module Type	Rated Power [W]	Nominal Power at 25th year [%]	Module efficiency at STC [%]
First Solar	FS_6445	Thin Film	445	86	17
First Solar	FS‐6390	Thin Film	390	86	15.76
Hanwha Qcells	Q.PEAK L‐G4.2 370	Monocrystalline	370	83.6	18.6
Jinko Solar	JKM 370M‐72	Monocrystalline	370	80.2	18.66
Longi Solar	LR6‐72 PE 370M	Monocrystalline	370	80.7	19.1

The typical lifetime of the selected models is 25 years according to their respective datasheets published by the manufacturers. The nominal power output percentage at 25th year is mentioned in the datasheet of the module. The theoretical calculation results for a capacity of 50 MW shows that First Solar with thin film technology generates 43 MW of AC power at 25th year and Hanwha Qcells provide 41.8 MW among the selected PV modules. In practical application, these values vary due to configuration, losses, location of site, conversion efficiency, etc. The power output and energy injected into the grid per year is evaluated using PVSyst for the selected modules.

### 3.4 Simulation Using PVSyst

The real time measurements and data are generated for the selected PV modules using PVSyst. The project design feature of the software for grid connected system is mainly utilized for simulation.

#### 3.4.1 Databases

The meteorological data for the location of Sepang is required to carry out simulation. However, the data base contains few meteorological station data for geographical sites like Kuala Lumpur, Kota Bharu, etc. The meteo file for Sepang is generated within PVSyst using Kuala Lumpur as base data and 17% of the satellite data. The meteo file is then exported and deployed for further analysis. The details for the geographical location of Sepang are as described in **Table** **2**.

**Table 2:** Meteonorm data for Sepang

PVSyst 6.8.1		
Databases		
Parameter	Data	
Geographical site	Sepang	
Country	Malaysia	
Region	Asia	
Source	Meteonorm 7.2 (1986–2005), Sat = 17%	
Latitude	2.6948	°
Longitude	101.7485	°
Altitude	21	m
Time Zone	8	GMT

**Figure 1:**
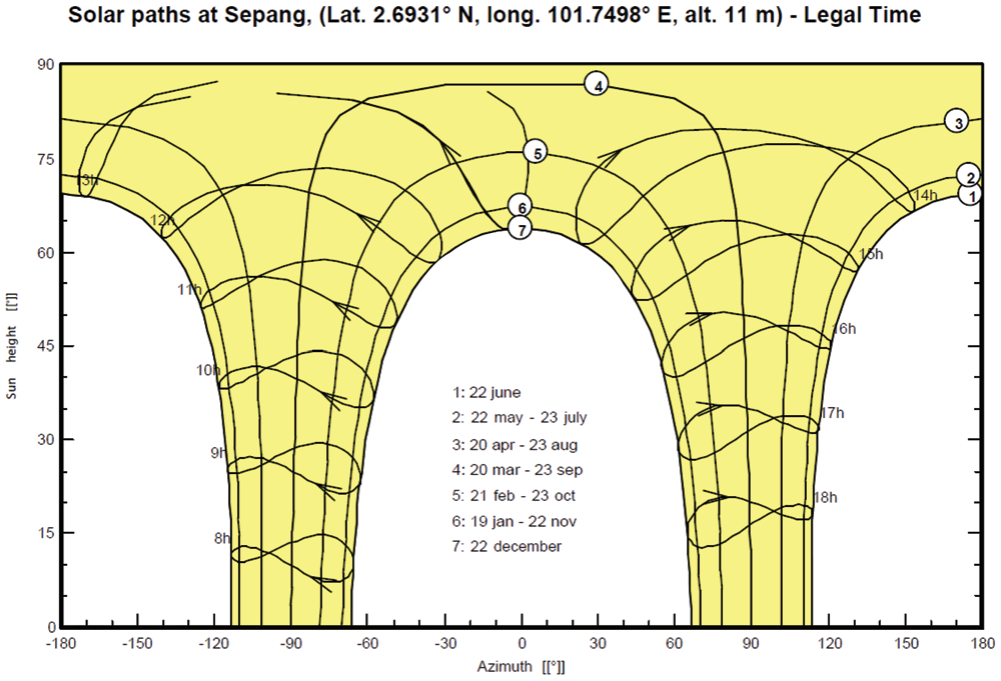
Sunpaths at Sepang, Selangor.

The sun path diagram for Sepang is generated from the preliminary design feature of PVSyst. The sun path obtained is given below in **Figure** **1**.

#### 3.4.2 Project Design for Grid Connected System

This section deals with main design parameters used to calculate the yearly energy generation and the energy injected into the power grid. The values and related parameters are listed below in **Table** **3**.

**Table 3:** Input parameters for project design of grid connected system

PVSyst 6.8.1	
Project Design for Grid Connected	
Project's Designation	
Parameter	Data
File Name	LSS_Selangor
Project's Name	Selangor 50MW
Site File	Sepang_MN72.SIT
Meteo File	Sepang_MN72_SYN.MET

System Variant	
Parameter	Data
Variant n°	VC1 Hanwha Qcells

Input Parameters—Orientation	
Parameter	Data
Field type	Fixed Tilted Plane
Plane Tilt	5°
Azimuth	0°
Optimization	Yearly irradiation yield

Input Parameters—System	
Parameter	Data
Planned Power	50 000 KWp
PV module	user defined
Inverter	user defined

Input Parameters—Detailed losses	
Parameter	Data
Thermal parameter	Free mounted modules with air circulation
Ohmic Losses	DC losses—1.5% at STC
	AC losses—0.5% at STC
	Transformer—default at STC
Module quality‐LID‐Mismatch	Default
Soiling loss	Default
Ageing	degradation factor as per data sheet of module

Input Parameters—Energy Management	
Parameter	Data
Power factor	0.85 power factor lagging
Inverter Pnom	KW

The tilt angle and azimuth angle of the system were selected as 5° and 0° respectively based on optimum design for the selected geographical location of Malaysia. Ahmed et al. confirmed the best configuration for tilt angle and azimuth angle in Malaysia through their research tilted “An Assessment of the Solar Photovoltaic Generation Yield in Malaysia using Satellite Derived Datasets.”^25^

The SMA inverters featuring inbuilt MPPT technique were connected to the PV modules in all cases to obtain maximum power generation. Different models from various manufactures like Siemens were tested yet not suitable for the configured system. Some of these models gave a warning message that the inverter is strongly oversized or undersized. The description of inverters connected to each model of PV modules selected is listed below in **Table** **4**.

**Table 4:** Inverter model and details

PV Model	Inverter Manufacturer	Inverter Model	No. of Inverters
FS_6445	SMA	Sunny Central 2500‐EV	16
FS‐6390	SMA	Sunny Central 2200	18
Q.PEAK L‐G4.2 370	SMA	Sunny Central 2200	18
JKM 370M‐72	SMA	Sunny Central 2200	18
LR6‐72 PE 370M	SMA	Sunny Central 2200	18

The simulation results provide the annual energy generation and the yearly active energy injected into the grid network. The apparent energy to the grid at 0.85 power factor lagging is also presented in the report.

### 3.5 IEEE‐9 Bus Test Bed

**Figure 2:**
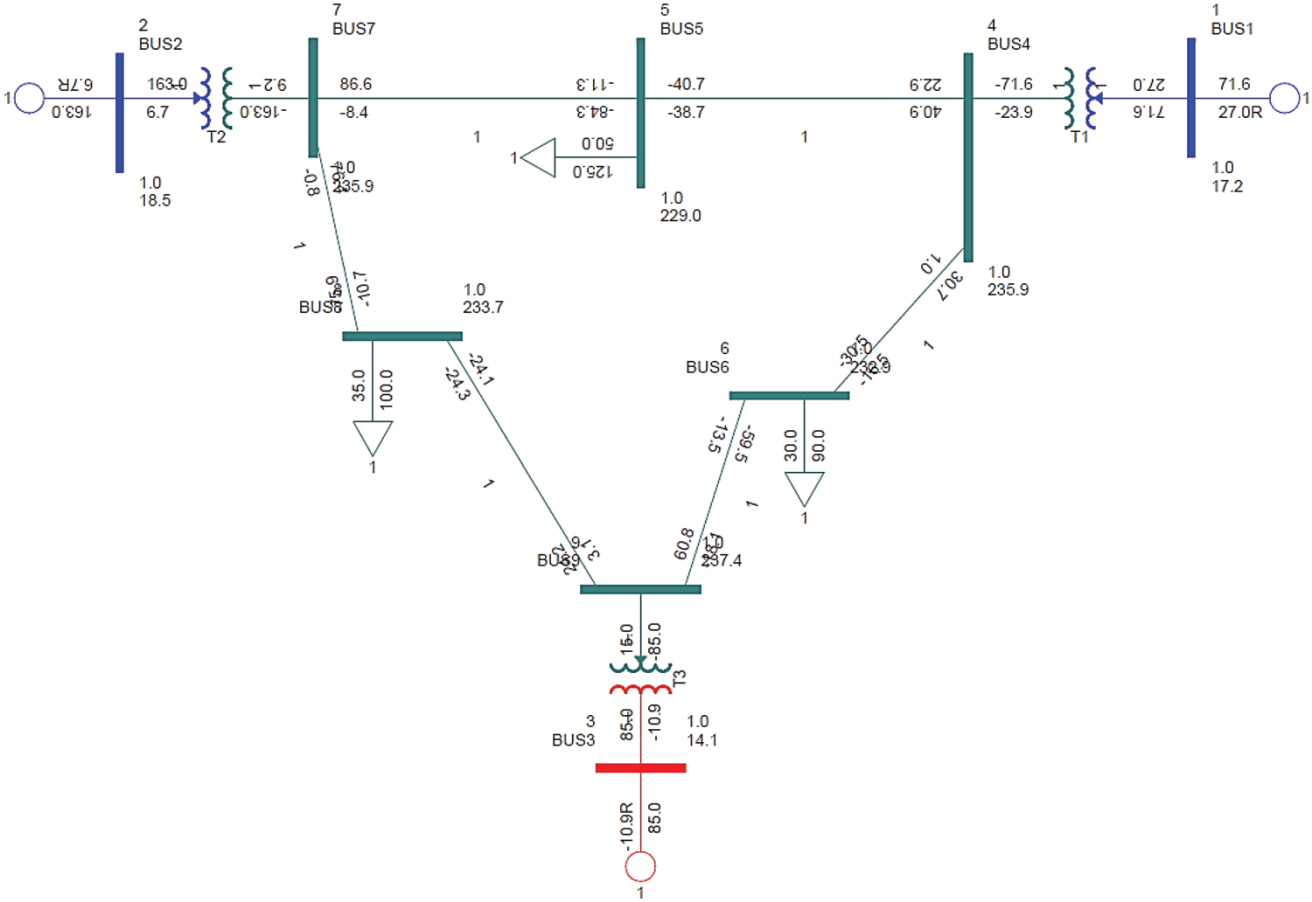
Single line diagram of IEEE‐9 bus test system.

The standard IEEE‐9 bus test system in accordance with the WSCC is adopted for this study. It is also known as the P.M. Anderson 9 bus and consists of 3 generators, 3 loads, and 9 buses. The base KV levels are 13.8, 16.5, 18, and 230 KV. Bus 5 (125 MW, 50 MVAR), bus 6 (90 MW, 30 MVAR), and bus 8 (100 MW, 35 MVAR) are the load buses. The **Figure** **2** below depicts the IEEE‐9 bus configuration.

The IEEE‐9 bus has the following transmission line parameters that require to be fed into the software to proceed with the analysis. **Table** **5** shows the values of resistance, reactance, and admittance of the standard IEEE‐9 bus test system.^26^

**Table 5:** IEEE‐9 bus line data for impedance and admittance

IEEE‐9 Bus Transmission Line Parameters			
Bus to Bus	Series Z [pu]		Shunt Y [pu]
	*R*	*X*	*B*
Transmission 1–4	0	0.0576	
Transmission 3–9	0	0.0586	
Transmission 2–7	0	0.0625	
Line 4–5	0.01	0.085	0.176
Line 4–6	0.017	0.092	0.158
Line 5–7	0.032	0.161	0.306
Line 6–9	0.039	0.17	0.358
Line 7–8	0.0085	0.072	0.149
Line 8–9	0.0119	0.1008	0.209

### 3.6 Solar PV Modeling in PSS/E

**Figure 3:**
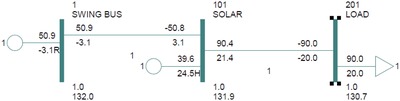
Simple solar generation bus with Swing bus and load.

The renewable energy modeling in PSS/E is different from models with conventional generators. The solar bus is connected to the swing bus and both buses have a base voltage level of 132 KV. The solar bus is connected to the IEEE‐9 bus test system using a 2 winding step‐up transformer to elevate the voltage level from 132 to 230 KV. The solar bus is connected to the swing bus as the latter acts as a reference bus for the solar bus and has a phase angle of zero degree. A simple model of solar renewable energy modeling with a load bus is shown in **Figure** **3** below.

Since PSS/E does not feature an option to model solar generation bus, it can be modeled as wind generator. The important parameter to consider is the control mode in wind data section of the machine data. The control mode selection is set to +, − Q limits based on WPF.

### 3.7 Simulation Using PSSE

A solar bus with a generation capacity of 39.6 MW which is the inverter output from PVSyst simulation was used as the model setup. The maximum capacity of generation was set to 50 MW with a base voltage of 132 KV.

#### 3.7.1 Solar Bus Configuration

The solar bus was modeled in three distinct configurations to study the effects of integration to the IEEE‐9 bus system. They are

- Case I ‐ Solar generation bus of 39.6 MW with a swing bus having base 132 KV.
- Case II ‐ Solar generation bus of 39.6 MW having base 132 KV without swing bus.
- Case III ‐ Solar generation bus of 39.6 MW having base 13.8 KV without swing bus.

Case II and Case III had the issue of islands during the course of simulation. These two cases were neglected and the simulations were carried out using case I configuration.

#### 3.7.2 Integration of Solar Bus with IEEE‐9 Bus

**Figure 4:**
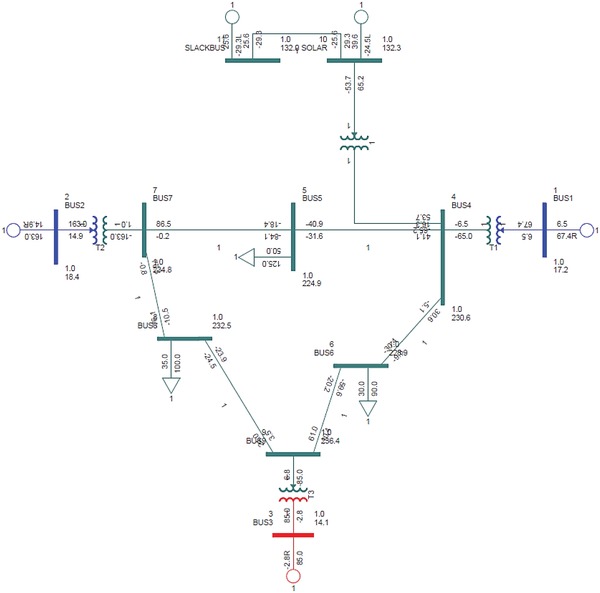
Solar generation bus interconnected to IEEE‐9 bus at bus 4.

The research was carried out to find the optimum configuration model for the integration of solar generation bus with the IEEE‐9 bus test system. The solar generation bus was interconnected to each of the buses from bus 4 to bus 9, one at a time on a case by case basis. The first setup was configured by connecting the solar generation bus to the bus 4 of the IEEE‐9 bus system using a step‐up transformer. The transformer steps up voltage level to 230 KV for interconnection to bus 4. The single line diagram of interconnection to bus 4 is depicted below in **Figure** **4**.

#### 3.7.3 Simulation Techniques

The simulation assessment was carried out for steady state analysis. The power flow analysis methods adopted were,

a. AC Contingency Analysis
b. *QV* Analysis

**Figure 5:**
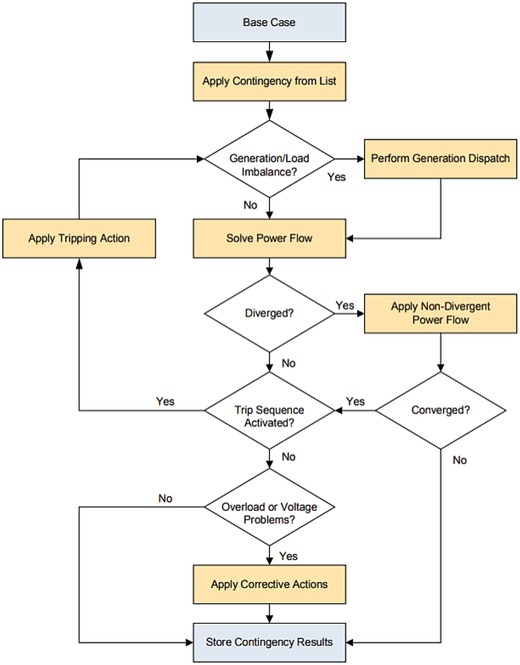
Flow chart for AC contingency procedure in PSS/E.

AC contingency is performed to assess the reliability of the power network. Contingency refers to the outage of a part of the network or the network itself. An outage of the power system component such as the generator or transmission line may cause overloading in transmission lines, deviations in bus voltages, power angle instability, or severe break down. Contingency analysis helps to provide reliability checks and corrective actions to the utilities for planning and operation of the network in real time. The computation procedure for contingency analysis using PSS/E^27^ is shown below in **Figure** **5**.

In this study, the main focus is to carry out the following AC contingency studies:

- AC Contingency Calculation (ACCC)
- Multilevel AC Contingency (MACC)

The AC Contingency is implemented for each case when the solar bus is connected to various buses of the IEEE‐9 bus test system. The files required are generated within PSS/E and the results are formulated in an excel sheet or text file. The behavior of the system is studied to identify the abnormality for a particular configuration scenario.

*QV* analysis is performed to plot the *QV* curve which determines the variations of the bus voltages with reactive power injection. *QV* curve is the correlation between the reactive power (*Q*) and voltage (*V*) for different active power (*P*) through the network.^28^ The *QV* curves for different buses can be plotted with values of reactive power MVAR and bus voltages from 0.9 to 1.1 pu. The reactive power demand can be calculated from the *QV* curves for worst case scenario and compensation devices of appropriate rating can be incorporated to improve system performance. *QV* Analysis is carried out to calculate the reactive power demand for each configuration when the solar bus is connected from bus 4 to bus 9 of IEEE‐9 bus system, on a case by case basis. The worst case contingency is noted and the reactive power demand for that particular bus is recorded. The performance of the worst configuration model can be improved with additional power electronic devices like reactive power compensation devices.

#### 3.7.4 Case I ‐ Solar Bus Connected to Bus 4 of IEEE‐9 Bus System

The first analysis done was with the solar bus integrated to the bus 4 on the IEEE‐9 bus test system. The solar generation bus was connected to the bus 4 by a two‐winding step‐up transformer to elevate the voltage levels to 230 KV. The schematic diagram of the solar bus interconnection to bus 4 in PSS/E is given in Figure 4.

The initial step was to solve the network using full Newton Raphson method. The Newton Raphson provides fast convergence and reduces computational time. The important parameter to be considered here is the convergence of the network. The met convergence tolerance must be verified to avoid blow up of the network.

The second step was to create the SUB, MON, and CON configuration files. Once these have been generated, the distribution factor data file (DFAX) was built using the same.

The third step was to perform contingency analysis to obtain AC contingency solution (ACCC). Furthermore, the multilevel AC contingency solutions were checked. The results were exported to excel sheets using the reports option under power flow tab.

Similarly the *QV* analysis was carried out and the reports were exported to excel sheets for further analysis. The results of the *QV* analysis were used to calculate the reactive power demand required for the optimal operation of the system at a bus voltage of 1.0 pu. The reactive power demand was calculated using the *QV* curves.

#### 3.7.5 Case I ‐ Configuration of Solar Bus Connected to Other Buses

The configuration used in case I was shifted from bus 4 to bus 5 of the IEEE‐9 bus. Similarly, the configuration change was done for each of the buses of IEEE‐9 bus from bus 6 to bus 9. The same procedure as in Section 3.7.4 was repeated for each configuration change and the results were exported to excel to determine the best configuration for the integration that satisfies the optimal power flow in the network. The worst case configuration was selected for the addition of reactive power compensators. This was done to improve the network performance of the worst case configuration.

#### 3.7.6 Case I ‐ Solar Bus Connected to Bus 8 of IEEE‐9 Bus with FACTS

**Figure 6:**
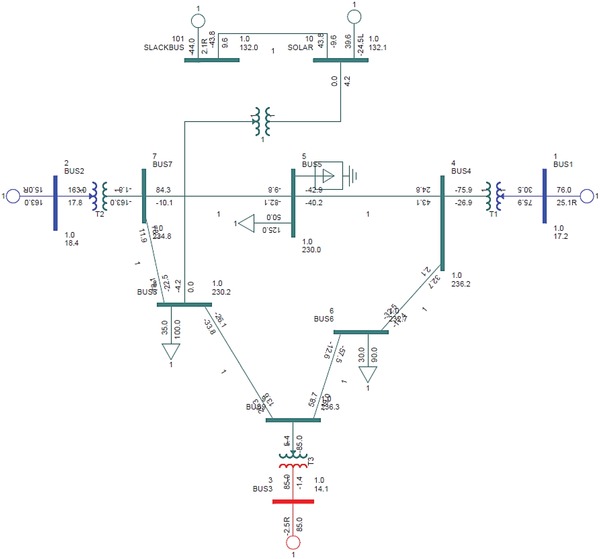
Solar generation bus connected to IEEE‐9 bus with FACTS.

The reactive power compensation was introduced to the network by employing FACTS. The reason for selecting FACTS is due to the availability of the component in the PSS/E dashboard and compensation is done for transmission lines. The reactive power compensation improves the bus voltages on lines and rectifies the worst case violations of single line AC contingency. A single line diagram of the modified network is depicted in **Figure** **6**.

The similar procedure as in Section 3.7.4 was followed to determine the changes in the load flow pattern. The results obtained were tabulated in excel and analyzed.

The same process was repeated to improve and analyze the integration of solar bus to bus 9 with FACTS. The AC contingency was performed to verify the appropriate rating of reactive power compensation device. The worst case violations in this specific case were tabulated.

## 4 Results and Analysis

The final results obtained from both PVSyst and PSS/E helps to determine the optimum configuration for the solar power generation plant and the interconnection to the IEEE‐9 bus system. The input parameters used to carry out simulation were kept almost identical to the realistic values used in the practical application for a similar system.

### 4.1 Results from PVSyst

The simulation was done for five different selected PV modules which includes monocrystalline and thin film cells. The monocrystalline modules of same power rating were opted for the comparative study. The thin film modules of similar specification were not available. Therefore, two modules of different power ratings in proximity with the monocrystalline modules were taken into consideration. The input parameters according to their respective datasheets were fed into PVSyst and other common parameters remained the same as specified in Section 3.4.2. The total AC power output from the inverter and the active energy injected into the grid were generated via the software. The results have been tabulated below in **Table** **6**.

**Table 6:** Annual energy generation with different modules

Model of PV Module	No of Modules	AC Power at Inverter Output [MW]	Energy at Inverter Output [GWh]
FS_6445	112 362	40	66.7
FS‐6390	128 204	39.6	67.5
Q.PEAK L‐G4.2 370	135 133	39.6	65.8
JKM 370M‐72	135 133	39.6	65.4
LR6‐72 PE 370M	135 133	39.6	66.7

**Figure 7:**
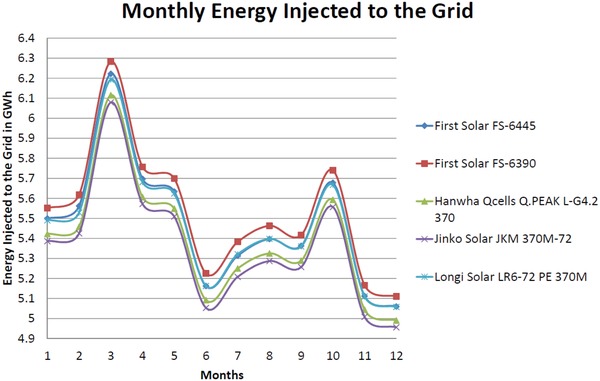
Monthly energy injected to grid network in GWh.

The total active energy injected into the grid per month for duration of one year is shown below in **Figure** **7**. From the chart in Figure 7, First Solar FS‐6390 proves to be the best module that generates maximum energy to the grid at 66.4 GWh per year. It is followed by First Solar FS‐6445 and Longi Solar LR6‐72 PE 370M which injects 65.7 GWh and 65.6 GWh per year respectively. Hanwha Qcells Q.PEAK L‐G4.2 370 generates 64.7 GWh per year grid injected energy while Jinko Solar JKM 370M‐72 stands at 64.3 GWh per year. In Malaysia, the maximum yield is received during the month of March and the least in December.

The higher energy generation by First Solar is due to higher rating of PV modules. However, being a thin film technology PV module that incorporates Cadmium Telluride (CdTe) they pose potential risk to the environment. The advantage of this module is number of modules required for the generation of 50 MW is comparatively less to other models. Among the monocrystalline technology, Longi Solar was leading in power generation profile with higher conversion efficiency. Monocrystalline is a well‐established technology and does not have negative effect on the environment. The performance characteristics and efficiency of monocrystalline modules are superior to the thin film modules.

### 4.2 Results from PSSE

The AC contingency analysis was performed for different configurations of solar bus connected to the IEEE‐9 bus. The worst case contingency violations, i.e., bus voltage (pu) drop below 0.95 or rise above 1.05 for each configuration was analyzed and tabulated below in **Table** **7**.

**Table 7:** Worst‐case violations from contingency analysis for different configurations

		Worst Case Violations—AC Contingency		
	Network Configuration	Contingency Applied	Bus Name	Bus Voltage [pu]
Reference Case	Solar bus not connected to system	Single line 4–5	Bus 5	0.839
		Single line 4–6	Bus 6	0.942
Option 1	Solar bus connected to bus 4	Single line 4–5	Bus 5	0.836
		Single line 4–6	Bus 6	0.94
Option 2	Solar bus connected to bus 5	Single line 4–6	Bus 6	0.942
Option 3	Solar bus connected to bus 6	Single line 4–5	Bus 5	0.838
Option 4	Solar bus connected to bus 7	Single line 1–4	Bus 4	0.933
		Single line 4–5	Bus 5	0.856
		Single line 4–6	Bus 6	0.935
Option 5	Solar bus connected to bus 8	Single line 1–4	Bus 4	0.939
		Single line 4–5	Bus 5	0.847
		Single line 4–6	Bus 6	0.939
Option 6	Solar bus connected to bus 9	Single line 1–4	Bus 4	0.925
		Single line 4–5	Bus 5	0.829
		Single line 4–6	Bus 6	0.924

The AC contingency is applied to the reference case where the solar bus is not connected to the IEEE‐9 bus system. The voltage level deviations were visible on bus 5 and bus 6 during single line contingency. The integration of solar bus to bus 4 for option 1 showed negligible voltage drops on bus 5 and bus 6 when compared to the reference case. The voltage increase was experienced on bus 5 for option 2 but remained within nominal limits. The voltage level on bus 6 was same as reference case for integration of solar bus to bus 5.

The connection of solar bus to bus 6 which was option 3 triggered voltage rises on bus 6 but bus 5 had the same voltage as reference case. The integration of solar bus in option 4, option 5, and option 6 to bus 7, 8, and 9 of IEEE‐9 bus test system affected voltage levels on several buses in each case. The impact was seen on bus 4, 5, and 6 of the IEEE‐9 bus according to the Table 7. However, the integration in each case increased voltage levels on bus 7, 8, and 9 respectively due to the point of interconnection but these were within specified nominal operating limits. The bus 5 and bus 6 are load buses and it is important to mitigate these violations to transfer maximum active power. Therefore, integration of solar bus for these configurations is recommended with additional power electronics like reactive power compensators. The application of reactive power compensators depends on the requirement of reactive power demand generated. This is determined by the *QV* curve generated from the *QV* analysis. The stable configuration from above results is option 1—integration of solar bus to bus 4 of IEEE‐9 bus system. The nonfeasible options like option 5 and option 6 were considered for performance improvement.

#### 4.2.1 Solar Bus Connected to Bus 8 of IEEE‐9 Bus System

The AC contingency worst case violations for integration to bus 8 (option 5) are as follows in **Table** **8**.

**Table 8:** Worst case violations for bus 8 interconnection to LSS PV (option 5)

		Worst Case Violations—AC Contingency		
	Network Configuration	Contingency Applied	Bus Name	Bus Voltage [pu]
Option 5	Solar bus connected to bus 8	Single line 1–4	Bus 4	0.939
		Single line 4–5	Bus 5	0.847
		Single line 4–6	Bus 6	0.939

**Figure 8:**
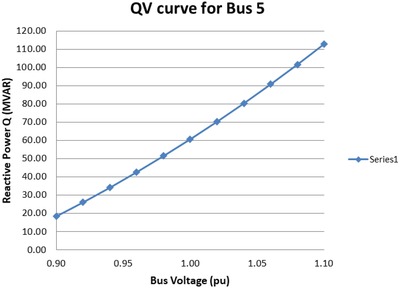
*QV* curve for bus 5 from Option 5 configuration.

The *QV* analysis for option 5 configuration is performed and the *QV* curves are generated. The *QV* curve is generated for bus 5 as this bus faced the most deviation in voltage level compared with bus 4 and 6. *QV* curve provides the amount of reactive power demand to be compensated. The *QV* curve for bus 5 is as shown below in **Figure** **8**.

The normal operation is possible if the bus voltage of bus 5 is elevated to the level of 1.0 pu. This is facilitated through reactive power compensation device that satisfies reactive power demand of 60.68 MVAR at bus 5.

#### 4.2.2 Solar Bus Connected to Bus 9 of IEEE‐9 Bus System

The worst case violations of bus voltages from AC contingency analysis for option 6 are listed in **Table** **9**.

**Table 9:** Worst case violations for bus 9 interconnection to LSS PV (option 6)

		Worst Case Violations—AC Contingency		
	Network Configuration	Contingency Applied	Bus Name	Bus Voltage [pu]
Option 6	Solar Bus connected to Bus 9	Single line 1–4	Bus 4	0.925
		Single line 4–5	Bus 5	0.829
		Single line 4–6	Bus 6	0.924

**Figure 9:**
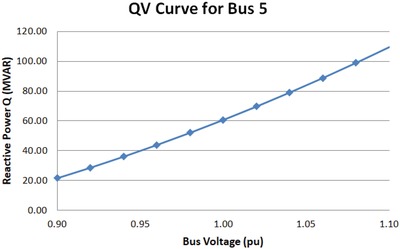
*QV* curve for bus 5 from Option 6 configuration.

In this case, again bus 5 is the most affected bus during single line contingency. Therefore, *QV* curve is generated from *QV* analysis to calculate the reactive power requirement of the system. The *QV* curve for bus 5 of option 6 is shown in **Figure** **9**.

The reactive power demand from the *QV* curve for normal operation of bus 5 at 1.0 pu operating voltage range is 60.66 MVAR. The FACTS device is connected to bus 5 in series to improve the voltage range on the transmission line.

#### 4.2.3 Solar Bus Connected to Bus 8 of IEEE‐9 Bus System with FACTS

The worst case violations of the solar bus integration to the bus 8 are rectified using FACTS device available within PSS/E. FACTS are a reactive power compensation device utilized for voltage regulation in this case. The justification to adopt FACTS on bus 5 is because bus 5 experiences most voltage drop during AC contingency analysis for option 5—normal configuration without FACTS. The reactive power demand of 60 MVAR is supplied by FACTS. The AC contingency analysis is performed for the modified system incorporating the FACTS device and the results are tabulated in **Table** **10** to study the improvement in the system.

**Table 10:** Comparison of bus voltage for option 5 with and without FACTS

Worst Case Violations—AC Contingency					
	Network Configuration	Contingency Applied	Bus Name	Bus Voltage [pu]	
				without FACTS	with FACTS
Option 5	Solar bus connected to bus 8	Single line 1–4	Bus 4	0.939	–
		Single line 4–5	Bus 5	0.847	–
		Single line 4–6	Bus 6	0.939	0.939

The voltage levels on bus 4 and 5 have increased and operate within desired limits when compared to the configuration without reactive power compensation. The Bus 6 voltage remains same in both cases but has a slight drop when measured against the reference case of IEEE‐9 bus test system. Therefore, the improved configuration of option 5 is more stable than the system without FACTS device in the network.

#### 4.2.4 Solar Bus Connected to Bus 9 of IEEE‐9 Bus System with FACTS

The AC contingency results for bus 9 with additional FACTS device installed on bus 5 are tabulated below in **Table** **11**.

**Table 11:** Comparison of bus voltage for option 6 with and without FACTS

	Worst Case Violations—AC Contingency				
	Network Configuration	Contingency Applied	Bus Name	Bus Voltage [pu]	
				without FACTS	with FACTS
Option 6	Solar bus connected to bus 9	Single line 1–4	Bus 4	0.925	–
		Single line 4–5	Bus 5	0.829	–
		Single line 4–6	Bus 6	0.924	0.924

A performance upgrade of the existing network is visible with the implementation of FACTS device supplying 60 MVAR. The violations on bus 4 and 5 are rectified and operate within specified voltage range. The bus voltage on bus 6 is 0.924 pu which has deviation of 1.8% from the reference case of IEEE‐9 bus test case where the voltage reading was 0.942 pu. However, the reliability of the overall system has improved and promotes higher active power transfer with reduced losses.

The best configuration for integration of solar bus to the IEEE‐9 bus test case is option 1 ‐connecting the solar PV bus to bus 4 which has similar performance characteristics as the reference system. The second option for integration to Bus 5 and 6 experiences increased voltage levels on bus 5 and 6 respectively but remains within normal operating limits. The integration to bus 7, 8, and 9 affects the voltage levels on bus 4, 5, and 6. Bus 5 encountered the maximum drop in voltage in all cases during single line contingency analysis. Therefore, configuration models like option 4, 5, and 6 can be implemented by addition of FACTS device on bus 5 to increase performance standards.

## 5 Conclusion

The thin film PV modules by First Solar with higher power rating generate higher yearly output. Hence, the number of modules required for the power plant will be considerably less when compared to the modules by monocrystalline technology for LSS. These can be installed in regions having limitations on land availability. The monocrystalline module by Longi Solar generates more output energy than their counterpart modules by Hanwha Qcells and Jinko Solar with the same ratings as specified by the manufacturer. It is also evident from the results that the energy generated by Longi Solar and First Solar is almost similar although the latter has a higher power rating. Monocrystalline modules are better than thin film modules using CdTe in terms of environmental aspects. The solar generation bus along with the swing bus having a base voltage level of 132 KV was integrated to the IEEE‐9 bus using a step‐up transformer to convert voltage levels to 230 KV. Swing bus was necessary with the solar generation bus to act as reference having phase angle zero to avoid islanding during contingency tests. The solar generation bus integration to bus 4 proved to be the best configuration because the deviations in bus voltages were minimal and the performance of the network resembled the actual IEEE‐9 bus test system configuration. The integration to bus 5 and 6 increased the voltages on bus 5 and 6 respectively but was within the operating limits. The configuration options 4, 5, and 6 (bus 7, 8, and 9) integration affected voltage range on bus 4, 5, and 6 for different single line contingency. Hence reactive power compensation was required to improve network performance to the desired limits. The reactive power was supplied by connecting FACTS in series to improve the bus voltages on transmission lines and changes in network performance were visible for option 5 and 6 with FACTS device.

## Conflict of Interest

The authors declare no conflict of interest.

## References

[1] National Renewable Energy Policy ‐ SEDA, https://www.seda.gov.my/policies/national-renewable-energy-policy-and-action-plan-2009 (accessed: August 2019).

[2] 2017 Malaysia Energy Statistics Handbook, Suruhanjaya Tenaga, Putrajaya, Malaysia 2017.

[3] MIDA Malaysian Investment Development Authority, https://www.mida.gov.my/home/8614/news/cypark-to-bid-for-100mw-solar-project-under-scheme-/ (accessed: August 2019).

[4] Intended Nationally Determined Contribution Of The Government Of Malaysia, 2015.

[5] Installation Of Grid‐Connected Photovoltaic (PV) System, 1st ed., Department Of Standards Malaysia, Putrajaya, Malaysia 2010, p. 10.

[6] P. Breeze , Solar Power Generation, Elsevier, Amsterdam 2016, pp. 71–80.

[7] S. Mekhilef , S. Abujarad , S. Ghazi , F. Shadman , Technical Issues of Grid Connected Renewable Energy Sources ‐ A New Areas of Research, 2014.

[8] S. Yasmeena , G. T. Das , Int. J. Energy Power Eng. 2015, 4, 22.

[9] W. Praiselin , J. Edward , Energy Procedia 2017, 117, 104.

[10] W. Suampun , Procedia Comput. Sci. 2016, 86, 301.

[11] P. S. R. Murty , Electrical Power Systems, Elsevier, Amsterdam 2017, pp. 479–526.

[12] L. Agnes Siau Wei , *master's thesis*, Universiti Tun Hussein Onn, Malaysia 2014.

[13] R. Kamdar , M. Kumar , G. Agnihotri , Electr. Comput. Eng.: Int. J. Vol. 2014, 3, 41.

[14] B. Sharma , R. Dahiya , J. Nakka , Electr. Power Syst. Res. 2019, 171, 1.

[15] A. Haidar , N. Julai , Energy Sustainable Dev. 2019, 50, 38.

[16] R. Zubo , G. Mokryani , H. Rajamani , J. Aghaei , T. Niknam , P. Pillai , Renewable Sustainable Energy Rev. 2017, 72, 1177.

[17] E. Youssef , R. M. El Azab , A. M. Amin , SoutheastCon 2015, IEEE, Piscataway, NJ 2015, p. 1.

[18] M. Merai , M. W. Naouar , I. Slama‐Belkhodja , E. Monmasson , Math. Comput Simul. 2019, 158, 344.

[19] A. Mohanty , A. Barik , Int. J. Mod. Eng. Res. 2019, 1, 666.

[20] E. Rakhshani , K. Rouzbehi , A. J. Sánchez , A. Tobar , E. Pouresmaeil , Energies 2019, 12, 1425.

[21] A. Narain , S. K. Srivastava , Int. J. Eng. Res. Technol. 2015, 4, 81.

[22] Grid Code For Peninsular Malaysia, 1st ed., Suruhanjaya Tenaga, Putrajaya, Malaysia 2016, pp. 168–186.

[23] Solar resource maps of Malaysia, https://solargis.com/maps-and-gis-data/download/malaysia (accessed: August 2019).

[24] Request For Proposal (RFP) For The Development Of Large Scale Solar Photovoltaic (LSS PV) Plants In Peninsular Malaysia And Sabah For Commercial Operation In 2017–2018 ‐ Announcement Of Shortlisted Bidder, 1st ed., Suruhanjaya Tenaga, Putrajaya, Malaysia 2017.

[25] T. Ahmed , S. Mekhilef , R. Shah , N. Mithulananthan , Int. Energy J. 2019, 19, 61.

[26] K. B. McGlashan , An Economic Analysis of Injecting Energy Storage into Power Systems Containing Renewables. 2017.

[27] PSS E ‐ Siemens, https://studylib.net/doc/18212045/pss-e—siemens (accessed: August 2019).

[28] M. Gupta , S. S. Matharu , Int. J. Eng. Res. Appl. 2018, 8, 1.

